# World Health Organization priority antimicrobial resistance in *Enterobacterales, Acinetobacter baumannii, Pseudomonas aeruginosa, Staphylococcus aureus* and *Enterococcus faecium* healthcare-associated bloodstream infections in Brazil (ASCENSION): a prospective, multicentre, observational study

**DOI:** 10.1016/j.lana.2025.101004

**Published:** 2025-01-30

**Authors:** Laura C. Antochevis, Camila M. Wilhelm, Beatriz Arns, Daniel Sganzerla, Letícia O. Sudbrack, Thais C.R.L. Nogueira, Ricardo D. Guzman, Amanda S. Martins, Daniela S. Cappa, Ândrea C. dos Santos, Joseani C. Pascual, Vitor Hugo Perugini, Eliana C. Vespero, Maria Helena P. Rigatto, Dariane C. Pereira, Larissa Lutz, Robson S. Leão, Elizabeth A. Marques, Danielle M. Henrique, André A.M. Coelho, Lígia L. Frutuoso, Erika E. de A Sousa, Luiz F. Abreu Guimarães, Adriana L.P. Ferreira, Anna Carla Castiñeiras, Marcelle D. Alves, João Paulo Telles, Carolina H. Yamada, Francieli P. de Almeida, Evelyne S. Girão, Paulo C.P. de Sousa, Antonio G.N.D. de Melo, Elisa T. Mendes, Verônica de F.D. Rocha, Euclimeire da S. Neves, Marcello T. Ribeiro, Carlos Ernesto Ferreira Starling, Maura S. Oliveira, Jorge L.M. Sampaio, Andreza F. Martins, Afonso L. Barth, Alexandre P. Zavascki, ASCENSION Study Group, Jéssica Nesello dos Santos, Charles Francisco Ferreira, Tarsila Vieceli, Julival Ribeiro Fagundes, Raquel Nascimento Matias, Shisue Karina Katagiri, Olavo José Vicente Neto, Rafaela Kuczynski da Rocha, Claudia Maria Dantas de Maio Carrilho, Mila Muraro de Almeida, Heloisa da Silva Rosa, Valéria Paes Lima, Tazio Vanni, Simone Aranha Nouer, Elizabeth Mendes Alves, Jorge Luiz Nobre Rodrigues, André Jhonathan Dantas, Gyselle de Souza Rebouças, Jailton Santos de Oliveira

**Affiliations:** a Laboratório de Pesquisa em Resistência Bacteriana (LABRESIS), Hospital de Clínicas de Porto Alegre, Porto Alegre, Brazil; b Programa de Pós-graduação em Ciências Médicas, Universidade Federal do Rio Grande do Sul, Porto Alegre, Brazil; c Universidade do Sul de Santa Catarina, Tubarão, Brazil; d Infectious Diseases Service, Hospital de Clínicas de Porto Alegre, Porto Alegre, Brazil; e Infectious Diseases and Infection Control Service, Hospital Moinhos de Vento, Porto Alegre, Brazil; f Inova Medical Research, Porto Alegre, Brazil; g Núcleo de Controle de Infecção Hospitalar, Hospital de Base do Distrito Federal, Brasília, Brazil; h Hospital de Base do Distrito Federal, Brasília, Brazil; i Hospital São Lucas, Porto Alegre, Brazil; j Hospital Universitário, Universidade Estadual de Londrina, Londrina, Brazil; k Serviço de Diagnóstico Laboratorial, Hospital de Clínicas de Porto Alegre, Porto Alegre, Brazil; l Department of Internal Medicine, Universidade Federal do Rio Grande do Sul, Porto Alegre, Brazil; m Hospital Universitário Pedro Ernesto, Universidade do Estado do Rio de Janeiro, Rio de Janeiro, Brazil; n Hospital Universitário de Brasília, Brasília, Brazil; o Hospital Universitário Clementino Fraga Filho, Universidade Federal do Rio de Janeiro, Rio de Janeiro, Brazil; p Laboratório Weinmann - Grupo Fleury, Porto Alegre, Brazil; q Hospital Universitário Evangélico Mackenzie, Curitiba, Brazil; r Hospital Universitário Walter Cantídio, Universidade Federal do Ceará, Fortaleza, Brazil; s Faculdade de Medicina, Universidade Federal do Ceará, Fortaleza, Brazil; t Hospital PUC-Campinas, Campinas, Brazil; u Pós Graduação Ciências da Saúde, PUC-Campinas, Campinas, Brazil; v Instituto Couto Maia, Salvador, Brazil; w Hospital Lifecenter, Belo Horizonte, Brazil; x Hospital Sírio Libanês, São Paulo, Brazil; y Faculdade de Ciências Farmacêuticas, Universidade de São Paulo, São Paulo, Brazil

**Keywords:** Antimicrobial resistance, *Enterobacterales*, *Klebsiella pneumoniae*, *Acinetobacter baumannii*, *Pseudomonas aeruginosa*, *Staphylococcus aureus*, *Enterococcus faecium*, Healthcare-associated infections, Bloodstream infections, Carbapenem resistance, Methicillin resistance, Vancomycin resistance, Epidemiology

## Abstract

**Background:**

Carbapenem-resistant *Enterobacterales* (CRE), *Acinetobacter baumannii* (CRAB), *Pseudomonas aeruginosa* (CRPA), methicillin-resistant *Staphylococcus aureus* (MRSA) and vancomycin-resistant *Enterococcus faecium* (VRE) are listed by World Health Organization (WHO) as priority antimicrobial-resistant bacteria. Data on WHO Priority Antimicrobial resistance Phenotype (WPAP) bacteria from low- and middle-income countries are scarce. In this study, we investigated the occurrence of WPAP in healthcare-associated bloodstream infections (BSI) in Brazil, an upper-middle-income country in South America.

**Methods:**

ASCENSION was a prospective, multicentre, observational study conducted in 14 hospitals from four of five Brazilian regions. *Enterobacterales*, *A. baumannii*, *P. aeruginosa, S. aureus* and *E. faecium* BSIs in hospitalised patients were analysed. The primary outcome was the frequency of WPAP among all bacteria of interest. Secondary outcomes were incidence-density of bacteria isolates in hospitalised patients, WPAP proportions within bacterial species, and 28-day mortality. PCR for carbapenemase genes was performed in carbapenem-resistant Gram-negative bacteria.

**Findings:**

Between August 15, 2022, and August 14, 2023, 1350 isolates (1220 BSI episodes) were included. WPAP accounted for 38.8% (n = 524; 95% Confidence Interval 32.0–46.1) of all isolates, with CRE (19.3%) as the most frequent, followed by CRAB (9.6%), MRSA (4.9%), VRE (2.7%), and CRPA (2.4%). Incidence-density of all and WPAP isolates were 1.91 and 0.77/1000 patients-day, respectively. Carbapenem-resistant *Klebsiella pneumoniae* (CRKP) was the most common CRE, corresponding to 14.2% of all BSIs. *A. baumannii* isolates presented the highest proportion of WPAP (87.8%). Mortality rates were higher in patients with BSIs by WPAP than non-WPAP isolates. KPC (64.4%) was the predominant carbapenemase in CRE, followed by NDM (28.4%) and KPC + NDM co-production (7.1%). OXA-23 was the most frequent in CRAB.

**Interpretation:**

A high frequency of WPAP bacteria, particularly CRKP and CRAB, were found in healthcare-associated BSIs in Brazil, posing them as a major public health problem in this country.

**Funding:**

10.13039/501100003593National Council for Scientific and Technological Development, Brazil.

**Research in context**

**Evidence before this study**

Antimicrobial resistance (AMR) is a critical global health challenge, contributing to increased mortality and healthcare system burdens worldwide. Healthcare-associated bloodstream infections (BSIs) caused by World Health Organization (WHO) priority antimicrobial resistance phenotype (WPAP) bacteria are associated with high morbidity and mortality rates. Although the burden of healthcare-associated infections is higher in low- and middle-income countries (LMICs), data on AMR have been disproportionately represented by high-income ones. This gap has been identified as a priority for global AMR research by the WHO.

We searched PubMed from database inception to December 31, 2024, for relevant published articles using the search terms “carbapenem-resistant *Enterobacterales”* OR “carbapenem-resistant *Acinetobacter baumannii”* OR “carbapenem-resistant *Pseudomonas aeruginosa”* OR “methicillin-resistant *Staphylococcus aureu*s” OR “vancomycin-resistant *Enterococcus faecium”* OR “WHO priority list”, AND “bloodstream infection∗” OR “bacteremia” OR “bacteraemia” AND “Healthcare associated” OR “Hospital associated” OR “Hospital-acquired” OR “Nosocomial”. Most studies are single-centre studies with only one of the WPAP bacteria, most from high-income countries. One systematic review addressed the frequency of BSIs of WPAP in Europe. One study examined the incidence of healthcare-associated BSIs in intensive care units in hospitals from India. Two recent retrospective multicentre studies addressed the occurrence of WPAP. One assessed the cumulative incidence of WPAP in the United States of America, and another, the proportion of WPAP and attributable mortality in Chile.

**Added value of this study**

This study provides comprehensive data on the frequency, incidence density, and mortality associated with WPAP bacteria in healthcare-associated BSIs across 14 tertiary-care hospitals in Brazil, an upper-middle-income country. Our findings reveal a worrisomely high frequency of WPAP isolates, particularly carbapenem-resistant *Klebsiella pneumoniae* and carbapenem-resistant *A*. *baumannii*, which together accounted for nearly a quarter of all BSI isolates. Moreover, it showed that, despite its lower frequency, methicillin-resistant *S. aureus* was significantly associated with higher mortality rates. This study highlights that the occurrence of WPAP BSIs is importantly higher in intensive care units patients. KPC production was the commonest mechanism determining carbapenem resistance in *Enterobacterales*, although a high proportion of NDM- and KPC + NDM-producing isolates were found. OXA-23 carbapenemase accounted for most resistance in *A. baumannii* isolates, while NDM-producing isolates predominated in *P. aeruginosa*.

**Implications of all the available evidence**

The high rates of WPAP in healthcare-associated BSIs in Brazil pose a menacing threat to public health in this country, which may resemble the epidemiological situation of other LMICs, particularly in Latin America. Our findings highlight the urgent need for the effective implementation of national action plans, emphasizing a comprehensive approach that not only minimizes antimicrobial overuse but also prioritizes the prevention of healthcare-associated infections and the control of WPAP bacteria transmission.

## Introduction

Antimicrobial resistance (AMR) represents one of the greatest public health challenges and an obstacle to sustainable global development, directly impacting increased mortality and the burden on healthcare systems.^1^ In 2021, more than 1 million deaths were attributed to AMR, and projections indicate 1.91 million annual deaths by 2050 if effective measures are not implemented.^2^ In the Americas, nations with limited access to antibiotics and healthcare services experience the highest AMR age-standardised attributable mortality rates.^3^

Based on several criteria such as incidence, morbimortality, treatability, among others, carbapenem-resistant *Enterobacterales* (CRE), *Acinetobacter baumannii* (CRAB), *Pseudomonas aeruginosa* (CRPA), methicillin-resistant *Staphylococcus aureus* (MRSA) and vancomycin-resistant *Enterococcus faecium* (VRE) were considered by World Health Organization (WHO) as critical or high priority antimicrobial resistant bacteria.^4^ These bacteria are major pathogens worldwide, most frequently causing healthcare-associated infections, regardless of their susceptibility profile.^5^

It has been shown that healthcare-associated infections affect patients' overall safety and outcomes worldwide, with greater public health impact on low- and middle-income countries (LMICs).^6^ Among healthcare-associated infections, bloodstream infections (BSIs) have been associated with the highest morbimortality.^7^ Indeed, bloodstream infections (BSI) were the second most common infection syndrome causing death among patients, following lower respiratory tract infections. This was true for infections caused by both antimicrobial-resistant and non-resistant bacteria.^2^^,^^5^ Additionally, healthcare-associated BSI by WHO priority AMR phenotype (WPAP) bacteria have been associated with even higher mortality compared with their susceptible counterparts.^8^, ^9^, ^10^, ^11^, ^12^, ^13^

The combination of healthcare-associated infections and antimicrobial resistance represent persistent and evolving threats to healthcare systems,^14^ and despite the higher burden of healthcare-associated infections in LMICs, data from these regions are scarce, and estimations of AMR have been disproportionately represented by high-income countries,^2^^,^^8^^,^^15^ which may enshroud the impact of AMR as a global threat.^8^ This critical gap has been recognized by WHO, which recently listed the investigation of the prevalence, incidence and mortality of healthcare-associated infections by WPAP bacteria, especially in LMICs, as one of the AMR research priorities.^16^

Therefore, considering the global challenge represented by AMR, the high burden of healthcare-associated BSIs combined with limited data from LMICs, we assessed the occurrence of WPAP in *Enterobacterales, A. baumannii* complex*, P. aeruginosa, S. aureus* and *E. faecium* in healthcare-associated BSI in Brazil, an upper-middle-income country in South America.

## Methods

### Study design and participants

The Antimicrobial reSistanCE iN bloodStream Infections in hOspitalised patieNts (ASCENSION) was a prospective, multicentre, cohort study conducted in 14 general tertiary-care hospitals from four of five Brazilian regions, from August 15, 2022 to August 14, 2023. Hospitals were selected by convenience, expecting to approximate their proportion in the study to the proportion of the Brazilian regions’ populations where each one was located (details can be found in Supplementary Material, p3). Patients admitted to any hospital unit were included if they were ≥18 years-old and had a healthcare-associated BSI for one of the following bacteria of interest: *Enterobacterales* species, *A. baumannii*, *P. aeruginosa*, *S. aureus,* or *E. faecium*. CRE, CRAB, CRPA, MRSA and VRE were classified as WPAP and their susceptible counterparts as non-WPAP bacteria. A healthcare-associated BSI was defined by the presence of at least one blood culture collected ≥48 h of hospitalisation that has been positive for any of the bacteria of interest. Meropenem resistance was assumed as carbapenem resistance. *Klebsiella pneumoniae* complex BSIs were also analysed separately from *Enterobacterales*. Other WPAP bacteria such as third-generation cephalosporin-resistant *Enterobacterales*, vancomycin-intermediate and -resistant *S. aureus* were not specifically evaluated in this study.

In patients with more than one episode of BSI caused by the same bacteria with the same AMR phenotype, only the first episode was considered in the analyses. If the patients had other BSI episodes by distinct bacteria or by the same bacteria but with distinct AMR phenotypes, either WPAP or non-WPAP, the additional episodes were included in the analyses and counted as another BSI episode (for examples, refer to Supplementary Material, p4), except for mortality analysis (explained below).

The institutional review boards of all hospitals approved the study. The informed consent was waived; however, only deidentified data related to the bacterial isolate, date and unit of admission and outcome at discharge were allowed to be collected from hospital/laboratory records without patients’ consent (Supplementary Material, p7).

### Procedures

For each BSI episode, length of hospital stays before positive blood culture, intensive care unit (ICU) admission at BSI episode and the outcome 28 days after the BSI episode were retrieved from electronic medical records. The number of patients-day (only patients ≥18 years-old were accounted for) were retrieved from the informatic systems of each hospital.

All participant sites had automated blood culture systems and blood cultures were collected according to medical decisions (Supplementary Material, p5). Susceptibility to meropenem for Gram-negative bacilli, methicillin (cefoxitin disk) for *S. aureus* and vancomycin for *E. faecium* were determined at each hospital's laboratories by disc diffusion according to the European Committee on Antimicrobial Susceptibility Testing (EUCAST), and results were interpreted according to the 2022 and/or 2023 version breakpoints from this committee.^17^

All WPAP isolates recovered from patients from all hospitals were eligible to be sent to a central laboratory, where re-identification by Matrix-Assisted Laser Desorption/Ionization Time-of-Flight Mass Spectrometry (MALDI-ToF-MS) and carbapenemase genes evaluation by quantitative polymerase chain reaction (qPCR) followed by high-resolution melting were performed (Supplementary Material, p5).

### Outcomes

The primary outcome was the frequency (absolute and relative) of isolates presenting WPAP among all bacteria of interest. Secondary outcomes were incidence-density of WPAP bacterial isolates in hospitalised patients, frequency (absolute and relative) of WPAP within each bacteria of interest group (species or order), and 28-day mortality rates (Supplementary Material, p6). For mortality, in patients with more than one BSI episode, only the first episode was considered, except if the second or any following episode was by a WPAP isolate, when the later was considered. Additionally, only monomicrobial BSIs were analysed for this outcome.

### Sample size

The sample size was estimated considering a proportion of WPAP of 30%, a confidence interval of 95% and a margin of error (±2.5%), which resulted in 1291 bacteria of interest. Clustering by hospital and individual patients was not considered in the sample size calculation, only in the statistical analysis. The total number was slightly higher because all sites collected data during the entire period of activation, as approved by institutional review boards since it did not bring any harm to patients. Additional information on sample size is provided in Supplementary Material, p7.

### Statistical analysis

The proportion of WPAP among all bacteria of interest isolates was calculated by dividing the n of WPAP by the total number of all bacteria of interest isolates; and of WPAP among each bacteria of interest by dividing the number of WPAP by the total number of that given bacteria of interest. The respective 95% confidence intervals (CI) were calculated using cluster robust standard errors, accounting for clustering by the hospital of origin of the isolate and individual patient.^18^^,^^19^ These metrics were also calculated for isolates from ICU and non-ICU patients.

Incidence-density was calculated as the number of bacteria of interest isolated divided by the number of patients-day x 1000. The respective 95% CI was constructed assuming a Poisson distribution,^20^ using the number of patient-days of each hospital as the offset term. Incidence-density ratios were calculated to compare incidence-densities of WPAP in ICU and non-ICU patients. Additional information on statistical analysis are provided in Supplementary Material, p7.

Cox regression was used to estimate hazard ratios for mortality comparing WPAP and non-WPAP isolates whilst adjusting for hospitals of origin as a random effect. The Cox proportional hazards model was performed using the coxph function.^21^ Proportional hazards assumption were checked graphically and using the Schoenfeld test, analysing Schoenfeld, Deviance, and Martingale residuals. Patients discharged before 28 days from a BSI episode were considered alive for crude frequency descriptions and were censored in the Cox model. Survival curves were estimated using Kaplan–Meier method.

A p-value of 0.05 was considered statistically significant. No adjustments for multiple comparisons were performed; therefore, the results of secondary outcomes should be interpreted as exploratory. All statistical analyses were performed in R version 4.3.2.^22^

### Role of the funding source

The funder of the study had no role in study design, data collection, data analysis, data interpretation, and writing of the report.

## Results

**Fig. 1:**
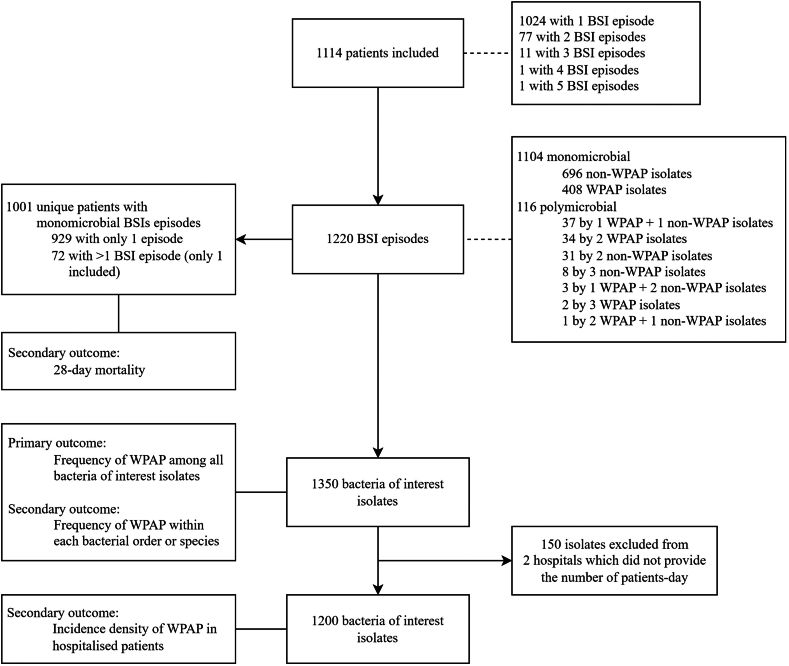
**Flowchart of the study**. WPAP, World Health Organization priority antimicrobial resistance profile; BSI, bloodstream infection. Of the 72 patients with >1 BSI episode, only the first episode was considered, except if the second or any following episode was by a WPAP isolate, when the later was considered.

A total of 1114 unique patients presented 1220 BSIs episodes (116 [9.5%] were polymicrobial), which resulted in the recovery of 1350 bacteria isolates of interest (Fig. 1). Eleven hospitals recruited patients for 6 months, two for 5 months, and one for 3 months, totalling 79 hospitals-month period of recruitment (Supplementary Material, p3). *Enterobacterales* species were the most commonly recovered isolates, followed by *S. aureus*, *A. baumannii* complex, *P. aeruginosa* and *E. faecium* (Table 1). In ICU, *A. baumannii* was more frequent than *S. aureus* (Table 1). *K. pneumoniae* complex (43.5%) was the most common *Enterobacterales* species (Supplementary Material, p8).

**Table 1:** Frequencies of all bacteria of interest and WHO priority antimicrobial resistance phenotype isolates.

Bacteria of interest	All bacteria of interest isolates^a^		WPAP isolates^b^	
	n	% (95% CI)	n	% (95% CI)
All	1350		524	38.8 (32.0–46.1)
*Enterobacterales*	763	56.5 (52.3–60.7)	260	19.3 (15.4–23.8)
*Klebsiella pneumoniae*	332	24.6 (20.1–29.8)	192	14.2 (11.2–17.9)
*Acinetobacter baumannii*	147	10.9 (7.5–15.5)	129	9.6 (6.6–13.6)
*Pseudomonas aeruginosa*	120	8.9 (6.9–11.3)	32	2.4 (1.6–3.4)
*Staphylococcus aureus*	255	18.9 (15.5–22.8)	66	4.9 (3.3–7.1)
*Enterococcus faecium*	65	4.8 (3.4–6.7)	37	2.7 (1.9–4.0)
**ICU isolates**	495		250	50.5 (39.5–61.5)
*Enterobacterales*	277	56.0 (49.5–62.2)	126	25.5 (18.4–34.1)
*Klebsiella pneumoniae*	133	26.9 (22.8–31.3)	90	18.2 (13.9–23.4)
*Acinetobacter baumannii*	72	14.5 (9.7–21.3)	68	13.7 (9.4–19.7)
*Pseudomonas aeruginosa*	49	9.9 (7.6–12.8)	15	3.0 (1.9–4.9)
*Staphylococcus aureus*	59	11.9 (8.0–17.4)	21	4.2 (2.1–8.3)
*Enterococcus faecium*	38	7.7 (4.6–12.6)	20	4.0 (2.3–7.0)
**Non-ICU isolates**	855		274	32.0 (27.5–37.0)
*Enterobacterales*	486	56.8 (52.0–61.6)	134	15.7 (13.0–18.8)
*Klebsiella pneumoniae*	199	23.3 (18.2–29.3)	102	11.9 (9.3–15.2)
*Acinetobacter baumannii*	75	8.8 (6.1–12.5)	61	7.1 (5.0–10.1)
*Pseudomonas aeruginosa*	71	8.3 (6.0–11.4)	17	2.0 (1.2–3.4)
*Staphylococcus aureus*	196	22.9 (19.1–27.3)	45	5.3 (3.7–7.4)
*Enterococcus faecium*	27	3.2 (2.7–3.7)	17	2.0 (1.5–2.7)

WPAP, World Health Organization priority antimicrobial resistance phenotype; NA, not applicable; ICU, intensive care unit.

a Bacteria of interest: *Enterobacterales*, *Acinetobacter baumannii*, *Pseudomonas aeruginosa*, *Staphylococcus aureus* and *Enterococcus faecium*. *Klebsiella pneumoniae* was the most common carbapenem-resistant *Enterobacterales* species and it was also analysed separately.

b Proportion among all 1350 bacteria of interest.

Of the 1350 bacteria of interest isolates, a total of 524 (38.8%, [95% CI 32.0–46.1]) were WPAP bacteria (Table 1). These 524 WPAP isolates were recovered from 485 BSI episodes, which occurred in 458 unique patients. 493 (40.4%, [95% CI 33.3–47.9]) WPAP of 1221 bacteria of interest isolates and 31 (24.0% [95% CI 16.4–33.8]) WPAP of 129 bacteria of interest isolates were recovered from patients admitted at public and private hospitals, respectively. The length of hospital stay before isolate recovery was significantly higher in WPAP than non-WPAP ones, except for CRAB and VRE isolates (Supplementary Material, p9). CRE were the WPAP bacteria with the highest frequency among all isolates, followed by CRAB, MRSA, VRE and CRPA, with similar order in both ICU and non-ICU isolates (Table 1). Carbapenem-resistant *K. pneumoniae* complex (CRKP) was the most common species (73.8%) among the CRE isolates (Supplementary Material, p8).

**Fig. 2:**
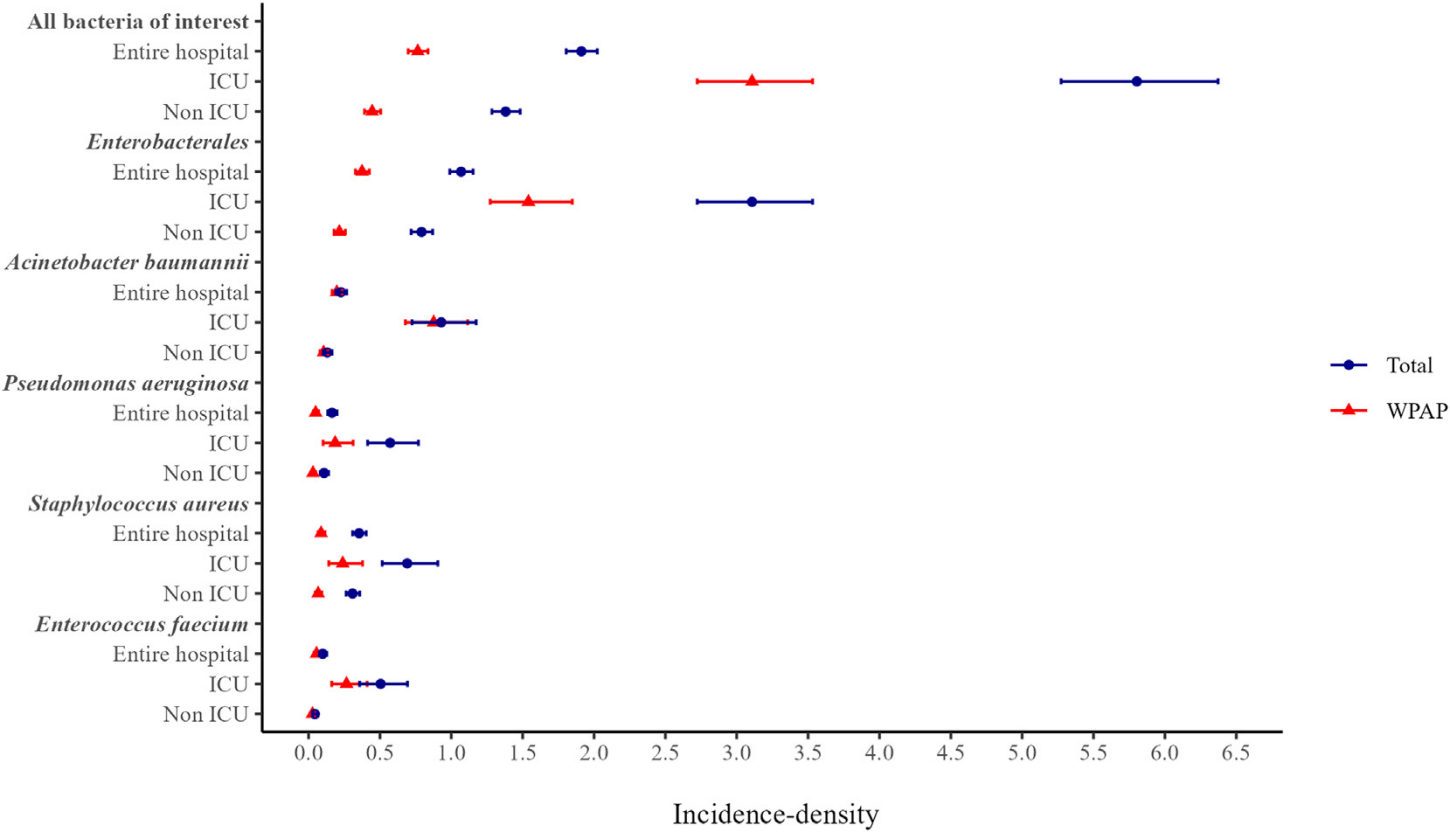
**Incidence-density of all bacteria of interest and WHO priority antimicrobial resistance phenotype (WPAP) bacteria in bloodstream infections in hospitalised patients**. Incidence-densities per 1000 patients-day (95% Confidence Interval) of all bacteria of interest in the entire hospital (EH), intensive care unit (ICU) and non-ICU patients are 1.91 (1.81–2.02), 5.80 (5.27–6.37) and 1.38 (1.29–1.48), respectively; all WPAP in EH, ICU and non-ICU = 0.77 (0.70–0.84), 3.11 (2.72–3.53) and 0.45 (0.39–0.51), respectively. Incidence-densities, including those of carbapenem-resistant *Klebsiella pneumoniae* separately, are shown in Supplementary Material, p10.

A total of 1200 bacteria of interest isolates occurring in 627,779 patients-day in the entire hospital (437 isolates in 75,302 ICU patients-day and 763 isolates in 552,477 in non-ICU patients-day) were included in the incidence-density analysis. The incidence-density of all bacteria was 1.91/1000 patients-day (95% CI 1.81–2.02): 5.80 (95% CI 5.27–6.37) and 1.38/1000 patients-day (95% CI 1.29–1.48) in ICU and non-ICU patients, respectively (Fig. 2, Supplementary Material, p10). The incidence-density of WPAP isolates was 0.77/1000 patients-day (95% CI 0.70–0.84): 3.11/1000 patients-day (95% CI 2.72–3.53) and 0.45/1000 patients-day (95% CI 0.39–0.51) in ICU and non-ICU patients, respectively (Fig. 2, Supplementary Material, p10). Incidence-densities of all WPAP and each WPAP bacteria were higher in ICU than in non-ICU episodes (Fig. 2, Supplementary Material, p10).

**Fig. 3:**
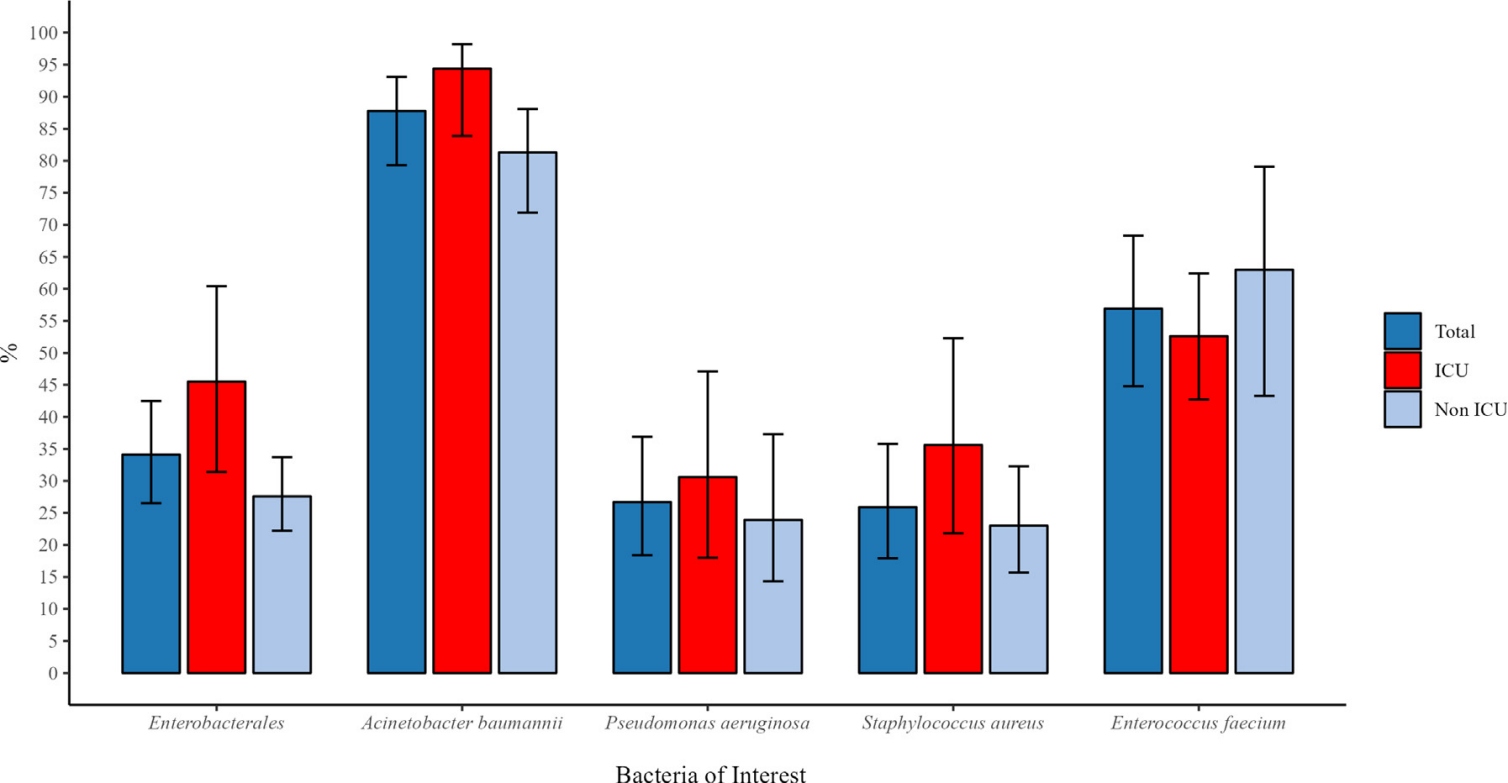
**Proportion of WHO priority antimicrobial resistance phenotype within each bacteria of interest**. Proportion (95% Confidence Interval [CI]) of carbapenem-resistant *Enterobacterales*: Total = 34.1 (95% CI 26.1–42.5), Intensive Care Unit (ICU) isolates = 45.5 (31.4–60.4), non-ICU = 27.6 (22.2–33.7); carbapenem-resistant *A. baumannii*: Total = 87.8 (79.3–93.1), ICU = 94.4 (83.9–98.2), non-ICU = 81.3 (71.9–88.1); carbapenem-resistant *P. aeruginosa*: Total = 26.7 (18.4–36.9), ICU = 30.6 (18.0–47.1), non-ICU = 23.9 (14.3–37.3); methicillin-resistant *S. aureus*: Total = 25.9 (17.9–35.8), ICU = 35.6 (21.8–52.3), non-ICU = 23.0 (15.7–32.3); vancomycin-resistant *E. faecium*: Total = 56.9 (44.8–68.3), ICU = 52.6 (42.7–62.4), non-ICU = 63.0 (43.3–79.1). The proportions of carbapenem-resistant *Klebsiella pneumoniae* are shown in Supplementary Material, p11.

The highest proportion of WPAP was observed among *A. baumannii* complex, followed by *E. faecium, Enterobacterales, P. aeruginosa* and *S. aureus* isolates, with similar order for ICU and non-ICU isolates (Fig. 3, Supplementary Material, p11). The number of isolates by hospital with their respective proportion of WPAP are shown in Supplementary Material, p12.

**Fig. 4:**
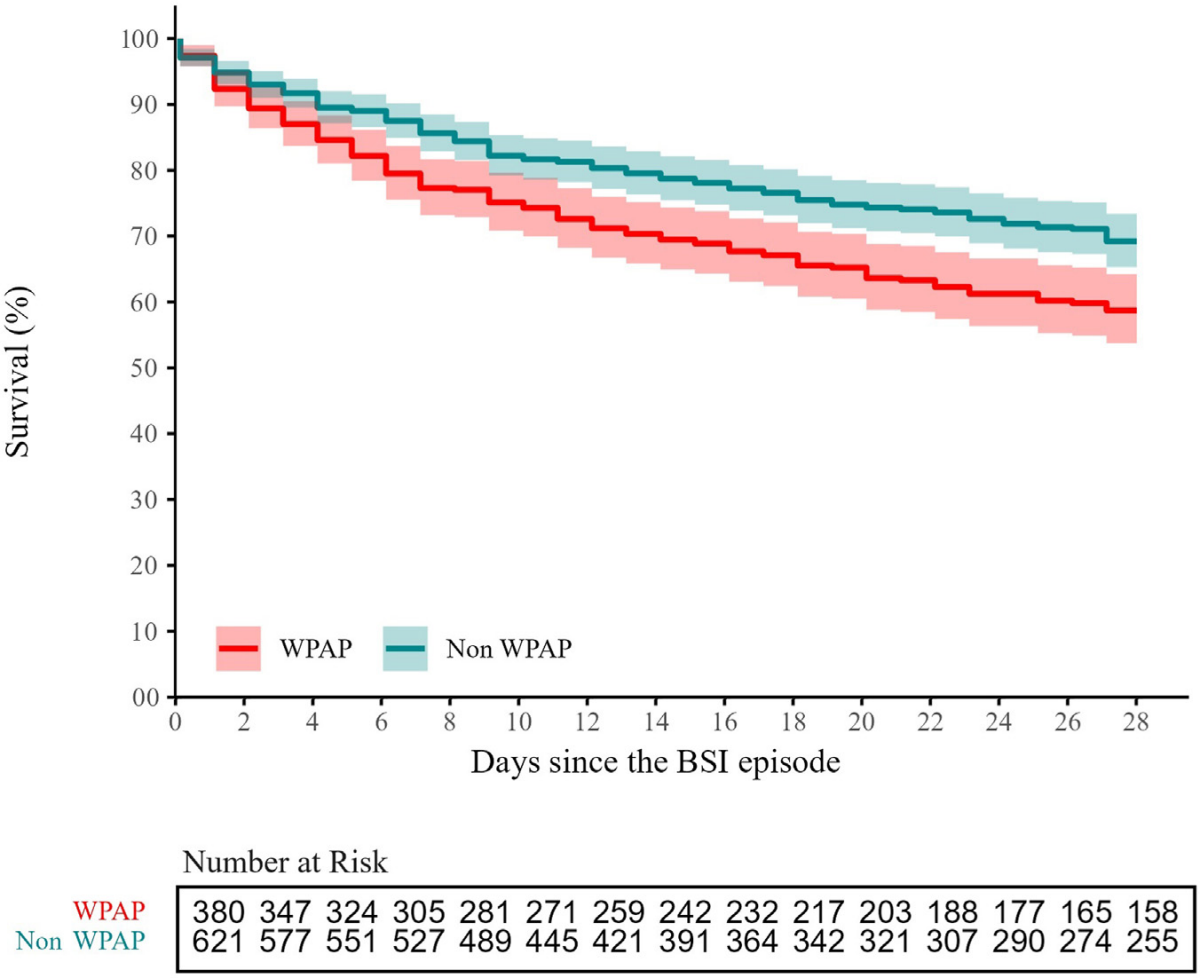
**Kaplan–Meier curve of 28-day mortality of unique patients with monomicrobial WHO priority antimicrobial resistance phenotype (WPAP) and non-WPAP bloodstream infections**. Of 1001 unique patients with monomicrobial bloodstream infections (BSIs), 331 (33.1) died within 28 days: 154 (40.5%) of 380 and 177 (28.5%) of 621 with BSI by WPAP and non-WPAP isolates, respectively; hazard ratio 1.36 (95% Confidence Interval 1.09–1.69), p = 0.007. Hazard ratio and p value were determined using a Cox proportional hazards model adjusted for hospital of origin. Shadow areas are 95% Confidence Intervals.

A total of 1001 unique patients with monomicrobial BSIs were included in 28-day mortality analysis: 380 (38.0%) were caused by WPAP and 621 (62.0%) by non-WPAP isolates. 331 (33.1%) patients died within 28 days: 154 (40.5%) of 380 and 177 (28.5%) of 621 of those with BSIs by WPAP and non-WPAP, respectively, hazard ratio 1.36 (95% CI 1.09–1.69), p = 0.007 (Fig. 4). The crude 28-day mortality and hazard ratios of each WPAP group are shown in Table 2.

**Table 2:** Hazard ratios for 28-day mortality per patient with monomicrobial bloodstream infections episodes.^a^

	Total n (%)/N^b^	WPAP n (%)/N^b^	non-WPAP n (%)/N^b^	HR (95% CI) for WPAP vs. non-WPAP^c^	p value^c^
**All**	331/1001 (33.1)	154/380 (40.5)	177/621 (28.5)	1.36 (1.09–1.69)	0.007
*Enterobacterales*	174/548 (31.8)	69/179 (38.5)	105/369 (28.5)	1.28 (0.94–1.76)	0.12
*Klebsiella pneumoniae*	90/242 (37.2)	60/142 (42.3)	30/100 (30.0)	1.38 (0.87–2.18)	0.17
*Acinetobacter baumannii*	42/98 (42.9)	42/91 (46.2)	0/7 (0)	NE	NE
*Pseudomonas aeruginosa*	42/87 (48.3)	11/21 (52.4)	31/66 (47.0)	1.27 (0.64–2.53)	0.50
*Staphylococcus aureus*	47/214 (22)	19/58 (32.8)	28/156 (17.9)	1.81 (1.01–3.24)	0.05
*Enterococcus faecium*	26/54 (48.1)	13/31 (41.9)	13/23 (56.5)	0.67 (0.29–1.55)	0.35

WPAP, WHO priority antimicrobial resistance phenotype; HR, Hazard ratio; CI, Confidence interval; NE, Not evaluated because of no event in one group.

a Only the first episode was included in the case of a patient with more than one episode, except if the second or any following episode was by a WPAP isolate, when the later was considered for analysis.

b n, number of deaths within 28-days; N, number of patients with bloodstream infection by bacteria in the line.

c Hazard ratios and p values were determined using Cox proportional hazards models adjusted for hospital of origin.

A total of 399 WPAP isolates sent to the central laboratory were viable for additional microbiological evaluation. The proportions of these 399 WPAP species submitted to further analysis were similar to those recorded in the study: 197 (49.4%; Supplementary Material, p13) CRE, 101 (25.3%) CRAB, 52 (13.0%) MRSA, 25 (6.3%) VRE and 24 (6.0%) CRPA. 194 (98.5%) CRE isolates carried a carbapenemase gene, either a *bla*_KPC_ (125/194, 64.4%), a *bla*_NDM_ (55/194, 28.4%), or both *bla*_KPC_ plus *bla*_NDM_ (14/194, 7.2%); while in three isolates (1.5% of 197) no carbapenemase gene was identified (Supplementary Material, p14). Of 101 CRAB isolates, 84 (83.2%) were positive only for *bla*_OXA-23_, three (3.0%) for *bla*_OXA-23+_*bla*_NDM_, while no carbapenemase was detected in the remaining. In CRPA, 16 (66.7%) of 24 isolates were positive for at least one carbapenemase gene: six for *bla*_NDM_ (one *bla*_NDM_ + *bla*_VIM_), five for *bla*_KPC_ (three *bla*_KPC_ + *bla*_GES_), three for *bla*_VIM,_ and two for *bla*_GES_.

## Discussion

This study showed a worrisome frequency of WPAP isolates (38.8%) in major bacteria causing healthcare-associated BSIs in Brazil. CRKP and CRAB were the most commonly found WPAP bacteria, corresponding together to 23.8% of all bacteria of interest. In ICU, the scenario was even more concerning, with WPAP accounting for half of all isolates, with CRKP and CRAB accounting for nearly a third (31.9%) of isolates from these units. Although it cannot be considered low, the frequency of other WPAP bacteria was less concerning. Indeed, the combined proportion of MRSA, VRE and CRPA isolates was exactly 10%, while *S. aureus, E. faecium* and *P. aeruginosa* together comprised 32.6% of all isolates.

Incidence-density of WPAP has not been previously evaluated in healthcare-associated BSIs. Indeed, a recent systematic review from European countries did not find any study addressing the incidence-density of WPAP.^23^ Pham et al. recently assessed the trends in the 30-day incidence of AMR in colonising or infecting isolates in US Veterans Affairs Healthcare System hospitals per 1000 hospital admissions.^24^ Although this metric adds more information than resistance proportions, this still did not account for the total number of patients at risk, as acknowledged by the authors.^24^ For comparative purposes, the incidence-density of bacteria of interest in ICU patients found in our study resembles those reported for all BSIs pathogens in adult ICUs from India, which ranged from 5.6 to 7.3/1000 patients-day.^25^ However, considering only WPAP bacteria, the incidence-density of 0.77/1000 patients-day observed in our study seems clearly concerning, because it is comparable to healthcare-associated BSIs rates (approximately 0.7 to 1.2/1000 patients-day from 2006 to 2018) by any pathogen in a study from Northern Denmark hospitals.^26^ Considering the lower number of patients-day in ICUs, the incidence-density adds relevant information on the impact of AMR in critically ill patients. In our study, the incidence-density of WPAP isolates (3.11/1000 patients-day) highly exceeded that observed in non-ICU patients (0.45/1000 patients-day), determining an incidence-density ratio of 7.00 (95% CI, 5.85–8.37). Notably, and highlighting the importance of incidence-density evaluation, despite the lower proportion of vancomycin resistance in *E. faecium* in ICU compared to non-ICU patients (52.6% and 63.0%), the incidence-density of VRE (9.79/1000 patients-day; 95% CI, 5.03–19.43) was the highest among each specific WPAP pathogen in ICU patients.

Most studies evaluating the proportion of WPAP reported lower proportions of WPAP among the bacteria of interest, particularly in Gram-negative isolates, than those found in our study.^12^^,^^13^^,^^23^^,^^24^ The higher proportions of AMR findings observed in our study performed in Brazil, an upper-middle-income country, are in accordance with previous studies that showed higher rates in LMICs than in high-income ones.^5^^,^^8^^,^^27^ However, it should be noted that our study was conducted in 2022/2023, while previous ones were performed some years before ours. The proportion of carbapenem resistance were higher (approximately 96% and 70% in *Acinetobacter* spp. and *Klebsiella* spp., respectively) in a meta-analysis of studies during the COVID-19 pandemics.^28^ However, in this same study, the occurrence of AMR was significantly lower in BSIs than in the respiratory tract (odds ratio 0.06, 95% CI 0.01–0.40); therefore, these proportions must be substantially lower if only BSI were considered.^28^

The increased 28-day mortality in WPAP patients is in line with the WHO studies that considered the higher mortality impact to define these bacteria of critical and high priority.^4^ Analysed separately, only patients with MRSA BSIs presented a statistically significant higher mortality compared with their susceptible counterparts, despite the lowest proportion of WPAP observed in *S. aureus* (25.9% were MRSA), highlighting the importance of MRSA as a major pathogen, from which the attributable mortality has been increasing worldwide since 1990.^2^ In Chile, MRSA also accounted for the highest attributable mortality among WPAP BSIs.^13^ CRE and CRKP BSIs had numerically higher mortality than those with BSIs by their susceptible counterparts, but differences were not statistically significant in our study. The highest difference in mortality was observed in patients with CRAB BSIs compared to no death in the few with carbapenem-susceptible *A. baumannii* BSIs. Regarding *P. aeruginosa*, remarkably high mortality rates were found in patients with BSIs by this pathogen, regardless of the carbapenem susceptibility profile, underpinning its relevance in healthcare-associated infections.

Noteworthy, mortality rates for CRE, CRAB and CRPA infections have been significantly higher in South and Central American countries than in other parts of the world.^29^, ^30^, ^31^ This alarming scenario may reflect shared health inequalities among these countries, including healthcare systems characteristics,^32^ but also restricted access to novel antimicrobials,^3^^,^^33^ which has been shown to be cost-effective to improve outcomes associated with AMR.^34^ Finally, the numerically higher crude mortality in the vancomycin-susceptible *E. faecium* compared to VRE BSIs relies on a low number of patients and should be interpreted with caution.

The *bla*_KPC_ was the predominant carbapenemase gene in CRE; however, a high proportion of *bla*_NDM_, particularly in CRKP, was noted, a finding previously reported after COVID-19 pandemics in Brazil^35^ and other regions.^36^ A concerning proportion of KPC/NDM-co-producing CRKP (8.2%) found in our study has also been reported in the post-pandemics period.^35^^,^^37^ OXA-23 carbapenemase continues to be the predominant mechanism in CRAB,^38^ and, in CRPA, metallo-carbapenemases were the most common mechanism of resistance, with NDM displacing the previously predominant SPM-1 carbapenemase in Brazil, as previously reported.^35^

Our study has limitations and data should be interpreted accordingly. We were unable to collect demographic and other patients' clinical data. Not having access to specific patients' individual data was a condition of institutional review boards for waiving the signed informed consent. We believe that potential losses determined by logistic limitations for having consent of all patients would affect the accuracy of the rates of WPAP isolates, which was our primary aim of this study; therefore, we opted for having the most accurate numbers in detriment to demographics and clinical data of patients. Second, all participating sites were tertiary-care hospitals selected by convenience, and, as ASCENSION was an exclusively academic cooperation network, they might represent those more engaged in research; hence, generalisation to all Brazilian hospitals must be done with caution. Third, our study did not consider the individual predictors of mortality since it was not our primary aim, and although our data allows an estimation of the impact of WPAP on the patients’ outcome, these analyses must be interpreted judiciously. Fourth, we did not evaluate the occurrence of other WPAP bacteria such as third-generation cephalosporin-resistant *Enterobacterales*, vancomycin-intermediate and -resistant *S. aureus*. Finally, not all WPAP isolates were viable at the central laboratory for additional analysis, and a more comprehensive molecular analysis was lacking; however, our data provide valuable information on major resistance mechanisms driving carbapenem-resistance rates in Gram-negative isolates in our study sample.

In summary, WPAP, particularly CRKP and CRAB, are major causes of healthcare-associated BSIs in Brazil. Despite its lower frequency, MRSA still represents a major problem owing to its impact on mortality. WPAP rates pose a menacing threat to public health in this country, which may resemble the epidemiological situation of other LMICs, particularly in Latin America. Future studies addressing not only the proportion of WPAP in each bacteria, but also their frequency relative to other pathogens as the incidence-density of WPAP infections are encouraged.

## Contributors

Conceptualisation: APZ. Funding acquisition: APZ and ALB. REDCap preparation: APZ, LCA and CFF. Data curation: APZ, LCA, DS and CMW. Data collection and local project administration: BA, LOS, ASM, CMDMC, MHPR, RSL, AAC, LFAG, JPT, ESG, ETM, VFDR, CEFS and MSO. Data collection, local microbiological storage and shipment: JLMS, BA, LOS, ASM, MHPR, RSL, AAC, LFAG, JPT, ESG, ETM, VFDR, CEFS, MSO, TCRLN, RDG, DSC, ÂCS, JCP, VHP, ECV, DPC, LL, EAM, DMH, LLF, EEAS, ALPF, ACC, MDA, CHY, FPA, PCPS, AGNDM, VFDR, ESN, MTR, Tarsila Vieceli, JRF, RNM, SKK, OJVN, RKR, MMA, HSR, VPL, Tazio Vanni, SAN, EMA, JLNR, AJD, GSR and JSO. Microbiological experiments at the central laboratory: LCA, CMW, AFM, ALB, DPC and JNS. Statistical analyses: DS. Access and data verification: APZ, LCA and DS. First draft writing: LCA and APZ. Interpretation of the results and critical review of the manuscript: all authors, including those listed in the ASCENSION Study Group. Decision to submit the manuscript: APZ and LCA (all authors agreed).

^‡^**ASCENSION Study Group**: Jéssica N dos Santos BSc, Charles Francisco Ferreira PhD (Laboratório de Pesquisa em Resistência Bacteriana, Hospital de Clínicas de Porto Alegre, Porto Alegre, Brazil; Departamento de Fisiologia, ICBS/UFRGS, Porto Alegre, Brazil); Tarsila Vieceli MD (Infectious Diseases Service, Hospital de Clínicas de Porto Alegre, Porto Alegre, Brazil); Julival R Fagundes MD, Raquel N Matias MD (Hospital de Base do Distrito Federal, Núcleo de Controle de Infecção Hospitalar do Hospital de Base do Distrito Federal, Brasília, Brazil); Shisue K Katagiri PharmD, Olavo J V Neto MSc, Rafaela K da Rocha MD (Hospital São Lucas, Porto Alegre, Brazil), Claudia M D de M Carrilho MD (Hospital Universitário de Londrina, Universidade Estadual de Londrina), Mila M. Almeida PhD, Heloisa S. Rosa BSc (Hospital Universitário Pedro Ernesto, Universidade do Estado do Rio de Janeiro, Faculdade de Ciências Médicas, Rio de Janeiro, Brazil), Valéria Paes Lima MD, Tazio Vanni MD (Hospital Universitário de Brasília, Brasília, Brazil), Simone Aranha Nouer MD, Elizabeth Mendes Alves BSc (Hospital Universitário Clementino Fraga Filho), Jorge Luiz Nobre Rodrigues PhD (Hospital Universitário Walter Cantídio da Universidade Federal do Ceará and Faculdade de Medicina da Universidade Federal do Ceará), André Jhonathan Dantas BSc (Hospital Universitário Walter Cantídio da Universidade Federal do Ceará), Gyselle de Souza Rebouças BSc (Faculdade de Farmácia da Universidade Federal do Ceará), Jailton Santos de Oliveira BSc (Instituto Couto Maia).

## Data sharing statement

Data are available upon a reasonable request to the corresponding author of the study.

## Declaration of interests

BA reports support for attending meetings from Merck Sharp & Dohme. VHP reports support for attending meetings from Merck Sharp & Dohm. JLMS reports consulting fees from Roche Diagnostics, honoraria for lecture from Eurofarma, and honoraria for manuscript writing from Merck Sharp & Dohm. Tarsila Vieceli reports a grant from GSK for a lecture, and support for attending meetings from Jannsen. All other authors declare no competing interests.

## Appendix A Supplementary data

Translated Summary Portuguese

Translated Summary Spanish

Supplementary Figures and Tables

List of ASCENSION Study Group
