# Supplementary material for: World Health Organization priority antimicrobial resistance in Enterobacterales, Acinetobacter baumannii, Pseudomonas aeruginosa, Staphylococcus aureus and Enterococcus faecium healthcare-associated bloodstream infections in Brazil (ASCENSION): a prospective, multicentre, observational study
Source: Lancet Reg Health Am. 2025 Jan 30;43:101004. doi: 10.1016/j.lana.2025.101004 (PMC11830303; doi:10.1016/j.lana.2025.101004)
Supplement: Translated Summary Portuguese [file mmc1.pdf]

__Editorial Disclaimer:__ This translation in Portuguese was submitted by the authors and we reproduce it as supplied. It has not been peer-reviewed. Our editorial processes have only been applied to the original abstract in English, which should serve as a reference for this manuscript.

Resumo

Introdução: *Enterobacterales* resistentes a carbapenêmicos (ERC), *Acinetobacter baumannii* a carbapenêmicos (ABRC), *Pseudomonas aeruginosa* a carbapenêmicos (PARC), *Staphylococcus aureus* resistente à meticilina (SARM) e *Enterococcus faecium* resistente à vancomicina (ERV) foram listados pela Organização Mundial da Saúde (OMS) como bactérias com resistência prioritária aos antimicrobianos. Dados sobre os Fenótipos Prioritários de Resistência Antimicrobiana da OMS (FPRO) em países de baixa e média renda são escassos. Neste estudo, investigamos a ocorrência do FPRO em infecções da corrente sanguínea (ICS) associadas a cuidados de saúde no Brasil, um país de renda média-alta da América do Sul.

Métodos: O ASCENSION foi um estudo observacional, prospectivo, multicêntrico, conduzido em 14 hospitais de 4 das 5 regiões brasileiras. Foram analisadas ICS causadas por *Enterobacterales*, *A. baumannii*, *P. aeruginosa*, *S. aureus* e *E. faecium* em pacientes hospitalizados. O desfecho primário foi a frequência de FPRO entre todas as bactérias de interesse. Os desfechos secundários foram densidade de incidência dos isolados bacterianos em pacientes hospitalizados, proporções de WPAP dentro de cada espécie bacteriana e mortalidade em 28 dias. PCR para genes de carbapenemase foi realizada em bactérias gram negativas resistentes a carbapenêmicos.

Resultados: Entre 15 de agosto de 2022 e 14 de agosto de 2023, 1350 isolados (1220 episódios de BSI) foram incluídos. FPRO representaram 38,8% (n=524; intervalo de confiança de 95%, 32,0-46,1) de todos os isolados, sendo ERC (19,3%) o mais frequente, seguido por ABRC (9,6%), SARM (4,9%), ERV (2,7%) e PARC (2,4%). As densidades de incidência de todos os isolados e de WPAP foram 1,91 e 0,77/1000 pacientes-dia, respectivamente. *Klebsiella pneumoniae* resistente a carbapenêmicos (KPRC) foi o ERC mais comum (14,2%). *A. baumannii* apresentou a maior proporção de FPRO (87,8%). As taxas de mortalidade foram mais altas em pacientes com ICS causadas por FPRO do que por não- FPRO. KPC (64,4%) foi a carbapenemase predominante em ERC, seguida por NDM (28,4%) e coprodução de KPC+NDM (7,2%). OXA-23 foi a mais frequente em ABRC.

Interpretação: Uma alta frequência de bactérias FPRO, particularmente KPRC e ABRC, foi encontrada em ICS associadas a cuidados de saúde no Brasil, representando um grave problema de saúde pública no país.

Financiamento: Conselho Nacional de Desenvolvimento Científico e Tecnológico (CNPq), Brasil.
