# Supplementary material for: World Health Organization priority antimicrobial resistance in Enterobacterales, Acinetobacter baumannii, Pseudomonas aeruginosa, Staphylococcus aureus and Enterococcus faecium healthcare-associated bloodstream infections in Brazil (ASCENSION): a prospective, multicentre, observational study
Source: Lancet Reg Health Am. 2025 Jan 30;43:101004. doi: 10.1016/j.lana.2025.101004 (PMC11830303; doi:10.1016/j.lana.2025.101004)
Supplement: Translated Summary Spanish [file mmc2.pdf]

Editorial Disclaimer: *This translation in Spanish was submitted by the authors and we reproduce it as supplied. It has not been peer-reviewed. Our editorial processes have only been applied to the original abstract in English, which should serve as a reference for this manuscript.*

Resumen

Introducción: *Enterobacterales* resistentes a carbapenémicos (ERC), *Acinetobacter baumannii* resistente a carbapenémicos (ABRC), *Pseudomonas aeruginosa* resistente a carbapenémicos (PARC), *Staphylococcus aureus* resistente a meticilina (SARM) y *Enterococcus faecium* resistente a vancomicina (ERV) han sido catalogados por la Organización Mundial de la Salud (OMS) como bacterias con resistencia antimicrobiana prioritaria. Los datos sobre los Fenotipos Prioritarios de Resistencia Antimicrobiana de la OMS (FPRO) en países de ingresos bajos y medianos son escasos. En este estudio, investigamos la ocurrencia de FPRO en infecciones del torrente sanguíneo (ITS) asociadas a la atención médica en Brasil, un país de ingresos medianos-altos en América del Sur.

Métodos: ASCENSION fue un estudio observacional, prospectivo y multicéntrico, realizado en 14 hospitales de 4 de las 5 regiones brasileñas. Se analizaron ITS causadas por *Enterobacterales*, *A. baumannii*, *P. aeruginosa*, *S. aureus* y *E. faecium* en pacientes hospitalizados. El desenlace primario fue la frecuencia de FPRO entre todas las bacterias de interés. Los desenlaces secundarios incluyeron densidad de incidencia de los aislados bacterianos en pacientes hospitalizados, proporciones de FPRO dentro de cada especie bacteriana y mortalidad a los 28 días. Se realizó PCR para genes de carbapenemasas en bacterias Gram negativas resistentes a carbapenémicos.

Resultados: Entre el 15 de agosto de 2022 y el 14 de agosto de 2023, se incluyeron 1350 aislados (1220 episodios de ITS). Los FPRO representaron el 38,8% (n=524; intervalo de confianza del 95%, 32,0-46,1) de todos los aislados, siendo ERC (19,3%) el más frecuente, seguido por ABRC (9,6%), SARM (4,9%), ERV (2,7%) y PARC (2,4%). Las densidades de incidencia de todos los aislados y de FPRO fueron de 1,91 y 0,77/1000 pacientes-día, respectivamente. *Klebsiella pneumonia*e resistente a carbapenémicos (KPRC) fue el ERC más común (14,2%). *A. baumannii* presentó la mayor proporción de FPRO (87,8%). Las tasas de mortalidad fueron más altas en pacientes con ITS causadas por FPRO en comparación con no-FPRO. KPC (64,4%) fue la carbapenemasa predominante en ERC, seguida por NDM (28,4%) y coproducción de KPC+NDM (7,2%). OXA-23 fue la más frecuente en ABRC.

Interpretación: Se encontró una alta frecuencia de bacterias FPRO, particularmente KPRC y ABRC, en ITS asociadas a la atención médica en Brasil, representando un grave problema de salud pública en el país.

Financiamiento: Consejo Nacional de Desarrollo Científico y Tecnológico (CNPq), Brasil.
