# Supplementary material for: World Health Organization priority antimicrobial resistance in Enterobacterales, Acinetobacter baumannii, Pseudomonas aeruginosa, Staphylococcus aureus and Enterococcus faecium healthcare-associated bloodstream infections in Brazil (ASCENSION): a prospective, multicentre, observational study
Source: Lancet Reg Health Am. 2025 Jan 30;43:101004. doi: 10.1016/j.lana.2025.101004 (PMC11830303; doi:10.1016/j.lana.2025.101004)
Supplement: Supplementary Figures and Tables [file mmc3.pdf]

Supplementary Material

World Health Organization priority antimicrobial resistance in *Enterobacterales*, *Acinetobacter baumannii*, *Pseudomonas aeruginosa*, *Staphylococcus aureus* and *Enterococcus faecium* healthcare-associated bloodstream infections in Brazil (ASCENSION): a prospective, multicentre, observational study

Summary

Supplementary Methods **3**

Supplementary Table 1. Characteristics of hospitals included in the study. 3

Supplementary Table 2. Examples of inclusion and exclusion criteria of bloodstream infections episodes in patients with more than one episode. 4

Bacterial identification at participant centres 5

Storage and shipment of bacterial isolates 5

Bacterial identification at central laboratory 5

Quantitative polymerase chain reaction (qPCR) followed by high-resolution melting for detection of carbapenemase genes 5

Supplementary Table 3. Outcomes definitions. 6

Protocol Registration 7

Sample Size 7

Statistical Analysis 7

Supplementary Results **8**

Supplementary Table 4. Frequency of Enterobacterales species and carbapenem resistance in isolates. 8

Supplementary Figure 1. Length of hospitalisation stay before recovery of each bloodstream infection isolates. 9

Supplementary Table 5. Incidence-density of all bacteria of interest in hospitalised patients. 10

Supplementary Table 6. Proportion of WPAP among each Enterobacterales, Acinetobacter baumannii complex, Pseudomonas aeruginosa, Staphylococcus aureus and Enterococcus faecium isolates. 11

Supplementary Figure 2. Number of isolates and proportion of WPAP among each bacteria of interest by hospital. 12

Supplementary Table 7. Isolates that were identified as different species by MALDI-ToF at the central laboratory 13

Supplementary Table 8. Results of the quantitative multiplex polymerase chain followed by high-resolution melting analysis for carbapenemase genes* of carbapenem-resistant Enterobacterales species. 14

References **15**

Supplementary Methods

Supplementary Table 1. Characteristics of hospitals included in the study.

**Brazilian Region / Hospital (n=14)**	N of beds	N of ICU beds	Public Hospital	N of months (period of participation)	N of patients-day in the entire hospital	N of ICU patients-day	% of hospitals represented in the study / % of Brazilian population the Region % *
South							35·7% / 14·3%
A	836	43	Yes	6 (08/15/2022 to 02/15/2023)	142,415	9,702	-
B	497	97	No	6 (09/01/2022 to 03/01/2023)	69,652	10,183	-
C	400	70	No	6 (10/30/2022 to 04/30/2023)	48,409	10,585	-
D	450	94	Yes	5 (03/15/2023 to 08/15/2023)	59,259	9,317	-
E	475	116	No	6 (09/15/22 to 10/15/22 and 01/01/23 to 05/30/23)	67,503	10,056	-
Southeast							35·7% / 42·0%
A	300	18	Yes	6 (09/15/2022 to 03/15/2023)	32,753	3,354	-
B	400	112	Yes	6 (09/01/2022 to 03/01/2023)	NP	NP	-
C	325	19	No	6 (08/15/2022 to 02/15/2023)	NP	NP	-
D	450	60	No	3 (05/15/2022 to 08/15/2023)	34,047	3,939	-
E	213	30	No	5 (03/15/2023 to 08/15/2023)	18,984	4,373	-
Midwest							14·3% / 7·8%
A	700	86	Yes	6 (08/15/2022 to 02/15/2023)	69,715	9,289	-
B	200	39	Yes	6 (09/15/2022 to 03/15/2023)	24,800	3,274	-
Northeast							14·3% / 27·0%
A	130	30	Yes	6 (08/15/2022 to 02/15/202)	22,073	4,087	-
B	220	16	Yes	6 (08/15/2022 to 02/15/202)	27,589	2,458	-
North	NA	NA	NA	NA	NA	NA	0 / 8·9%

NA, not applicable; NP, not provided. * Proportion of population retrieved from 2022 data available in the "Instituto Brasileiro de Geografia e Estatística" (https://www.ibge.gov.br/ last accessed on August 23rd, 2022)

Supplementary Table 2. Examples of inclusion and exclusion criteria of bloodstream infections episodes in patients with more than one episode.

Example Patient	First Episode	Second Episode*	Third Episode †	Conduct	Reason
A	MSSA	MSSA	No	Only the first episode was included. Exclusion of the second episode.	Same bacterium with the same AMR phenotype
B	CS *E. coli*	CR *E. coli*	CR *E. coli*	Inclusion of the first and second episodes as distinct episodes. Exclusion of the third episode.	Inclusion of the second episode: same bacterium but distinct AMR profile. Exclusion of the third episode: same bacterium with the same AMR phenotype of the second episode.
C	MSSA + CRPA	CRPA	CSE	First and third episode included. Second episode excluded.	First episode is considered a polymicrobial episode. Second episode was excluded because the same bacterium with the same AMR phenotype has been included in the first episode.
D	CR *K. pneumoniae*	CR *E. cloacae*	No	Inclusion of both episode	Distinct bacterium with the same AMR phenotype. Both counted as distinct CRE episodes.

AMR, antimicrobial resistance; CS, carbapenem-susceptible; CSE, carbapenem-susceptible *Enterobacterales*; CRE, carbapenem-resistant *Enterobacterales*; CRPA, carbapenem-resistant *Pseudomonas aeruginosa*; MSSA, methicillin-susceptible *Staphylococcus aureus*. * Second episode necessarily occurring on a distinct day of the first episode either in the hospital admission or in another hospital admission. †Third episode necessarily occurring on a distinct day of the second episode either in the hospital admission or in another hospital admission.

Bacterial identification at participant centres

Isolates were identified at microbiology laboratory of each centre by automated biochemical tests, using VITEK® 2 (bioMerieux, Marcy-l'Etoile, France; seven sites), MicroScan Walkaway (Beckman Coulter, Atlanta, GA, USA; one site), or by matrix-assisted laser desorption ionisation time-of-flight mass spectrometry (MALDI-TOF-MS), with a VITEK® MALDI-TOF MS V3.0 MYLA® v 4.7.1 (bioMerieux, Marcy-l'Etoile, France) or a Microflex LT® V9.0 (Bruker Daltonics, Bremen, Germany; three sites) or both Vitek 2 and MALDI-ToF-MS (two centres).

Storage and shipment of bacterial isolates

WHO priority antimicrobial resistance phenotype (WPAP) isolates (plus meropenem- susceptible increased exposure [I]-Gram-negative bacteria) were stored on glycerol 15% at -70° to -20°C (two laboratories stored under refrigeration of 2-8°C) at each centre and further sent to the central laboratory of *Instituto Nacional de Pesquisa em Resistência Antimicrobiana* (https://inpra.com.br/) using swab containing Amies agar gel. Shipment to the central laboratory was performed every two or three months.

Bacterial identification at central laboratory

WPAP isolates were re-identified by MALDI-ToF-MS, using a Microflex LT mass spectrometer (Bruker, Germany).

Quantitative polymerase chain reaction (qPCR) followed by high-resolution melting for detection of carbapenemase genes

The bla_{KPC}, bla_{NDM}, $bla_{\text{OXA-48-like}}$, bla_{IMP}, bla_{GES}, bla_{VIM}, and $bla_{\text{SPM-1}}$ carbapenemase genes were evaluated in CRE, CRPA, and CRAB isolates by qPCR assay followed by high-resolution melting (adapted from Monteiro *et al*)[1] and $bla_{\text{OXA-23-like}}$ (only in carbapenem-resistant *Acinetobacter baumannii*) by conventional PCR.[2]

Supplementary Table 3. Outcomes definitions.

Outcome	Numerator	Denominator	Comments
Primary			
Frequency of WPAP among all bacteria of interest isolates	N of WPAP bacterial isolates	N of all bacterial of interest isolates	Frequency of all WPAP bacteria and each WPAP bacteria separately were calculated over the same denominator (all bacteria of interest)
Secondary			
Incidence-density of WPAP in hospitalised patients	N of WPAP bacterial isolates	N of patients-day	Two hospitals could not provide the number of patients-day and were excluded from the analysis of this outcome.
Proportion of WPAP within each bacteria of interest*	N of WPAP in a given bacteria of interest	N of that given bacteria of interest	Example: CRE over all *Enterobacterales*; MRSA over all *S. aureus*.
28-day mortality in monomicrobial BSIs	N of 28-day deaths	N of patients presenting monomicrobial BSI	28 days after the BSI episode.<br>In patients with >1 BSI episode, only the first was considered, except if the second or any following episode was by a WPAP isolate, when the later was considered.

WPAP, WHO priority antimicrobial resistance phenotype. CRE, carbapenem-resistant *Enterobacterales*; MRSA, methicillin-resistant *Staphylococcus aureus*. * Second episode necessarily occurring on a distinct day of the first episode either in the hospital admission or in another hospital admission. †Third episode necessarily occurring on a distinct day of the second episode either in the hospital admission or in another hospital admission.

Protocol Registration

The protocol was registered in the "Plataforma Brasil" (www.saude.gov.br/plataformabrasil) under the identification code CAAE: 54535521.4.1001.5327.

Sample Size

The initial sample size was estimated considering a proportion of WPAP of 30%, for a confidence interval of 95% and a margin of error (±1.5%). We also added a 20% increase in sample size for possible losses of registry and antimicrobial resistance profiles. The protocol population was 4688 bacterial isolates, and 15 sites recruiting for 6 months each one was expected to be necessary. During the study, we realised that such a restrict margin of error (±1.5%) was not necessary for such a study in a way that we could have a much lower number of isolates for an accurate estimate. In addition to the strict initial margin of error, the 20% increase for possible losses was not necessary, since no loss occurred in the conduction of the study.

The sample size was then re-estimated considering the same proportion of WPAP of 30%, a confidence interval of 95% and a margin of error (±2.5%), resulting in 1291 bacteria of interest.

Statistical Analysis

The 95% confidence intervals (CIs) for binomial proportions were calculated using a robust variance estimation approach to account for clustering at multiple levels (hospital of origin and individual patient). This method utilizes cluster-robust standard errors, which provide consistent standard error estimates even in the presence of within-cluster correlation. In R, this approach was implemented through the sandwich package, specifically using the vcovCL function, which computes heteroskedasticity- and cluster-robust covariance matrix estimators.[3,4]

For 95% CI of incidence-densities, a Poisson regression was used because we were working with aggregated data on patient-days per hospital, without having individual-level data on patient-days without infection. This approach is a widely accepted and standard method for analysing incidence density data, especially when dealing with rates of events over time across different clusters. By employing this method, we ensure that the CIs appropriately reflect the distribution of our data, even in the absence of individual patient-day data without infection, while accounting for the clustered structure of the study design.

Supplementary Results

Supplementary Table 4. Frequency of *Enterobacterales* species and carbapenem resistance in isolates.

**Enterobacterales**	All<br>n=763	% (95% CI)	CRE<br>n=260	% (95% CI)*
Klebsiella pneumoniae complex	332	43·5 (33·8 - 48·5)	192	73·8 (66·0 - 84·0)
Escherichia coli	145	19·0 (18·3 - 26·8)	3	1·2 (0·5 - 4·1)
Serratia marcescens	78	10·2 (4·5 - 12·1)	30	11·5 (2·5 - 16·8)
Enterobacter cloacae complex	56	7·3 (5·7 - 11·7)	7	2·7 (1·2 - 5·1)
Proteus mirabilis	45	5·9 (3·9 - 9·7)	5	1·9 (0·7 - 4·1)
Klebsiella oxytoca	21	2·8 (2·0 - 5·4)	3	1·2 (0·4 - 5·8)
Others†	86	11·3 (6·7 - 19·6)	20	7·7 (3·4 - 21·4)
**ICU isolates**	277		126	
Klebsiella pneumoniae	133	48·0 (41·3 - 54·8)	90	71·4 (58·6 - 81·6)
Serratia marcescens	42	15·2 (10·5 - 21·5)	21	16·7 (10·8 - 24·9)
Escherichia coli	37	13·4 (8·7 - 19·9)	1	0·8 (0·1- 6·4)
Enterobacter cloacae complex	16	5·8 (3·6 - 9·1)	2	1·6 (0·6 - 4·2)
Proteus mirabilis	15	5·4 (2·7 - 10·6)	3	2·4 (0·8 - 6·7)
Klebsiella oxytoca	5	1·8 (0·7 - 4·6)	1	0·8 (0·1 - 6·6)
Others	29	10·5 (7·7 - 14·2)	8	6·3 (3·7 - 10·8)
**Non-ICU isolates**	486		134	
Klebsiella pneumoniae complex	199	40·9 (33·8 - 48·5)	102	76·1 (66·0 - 84·0)
Escherichia coli	108	22·2 (18·3 - 26·8)	2	1·5 (0·5- 4·1)
Enterobacter cloacae complex	40	8·2 (5·7 - 11·7)	5	3·7 (1·8 - 7·5)
Serratia marcescens	36	7·4 (4·5- 12·1)	9	6·7 (2·5 - 16·8)
Proteus mirabilis	30	6·2 (3·9 - 9·7)	2	1·5 (0·3 - 7·3)
Klebsiella oxytoca	16	3·3 (2·0 - 5·4)	2	1·5 (0·4 - 5·8)
Others	57	11·7 (6·7 - 19·6)	12	9·0 (3·4 - 21·4)

CRE, carbapenem-resistant *Enterobacterales*; CI, Confidence interval., ICU, Intensive care unit. * % and 95%CI refers to the total of CRE (n=260). † *Enterobacter* spp. (n=17), *Morganella morganii* (n=16), *Klebsiella aerogenes* (n=10), *Citrobacter freundii* complex (n=10), *Providencia stuartii* (n=6), *Salmonella* spp. (n=5), *Citrobacter koseri* (n=4), *Citrobacter* spp. (n=3), *Pantoea agglomerans* (n=3), *Klebsiella variicola* (n=2), *Proteus penneri* (n=2), *Serratia liquefaciens* (n=2), *Citrobacter amalonaticus* (n=1), *Citrobacter youngae* (n=1), *Enterobacter aerogenes* (n=1), *Enterobacter asburiae* (n=1), *Klebsiella* spp. (n=1), *Kluyvera ascorbata* (n=1), *Proteus* spp. (n=1), *Proteus vulgaris* (n=1), *Providencia rettgeri* (n=1).

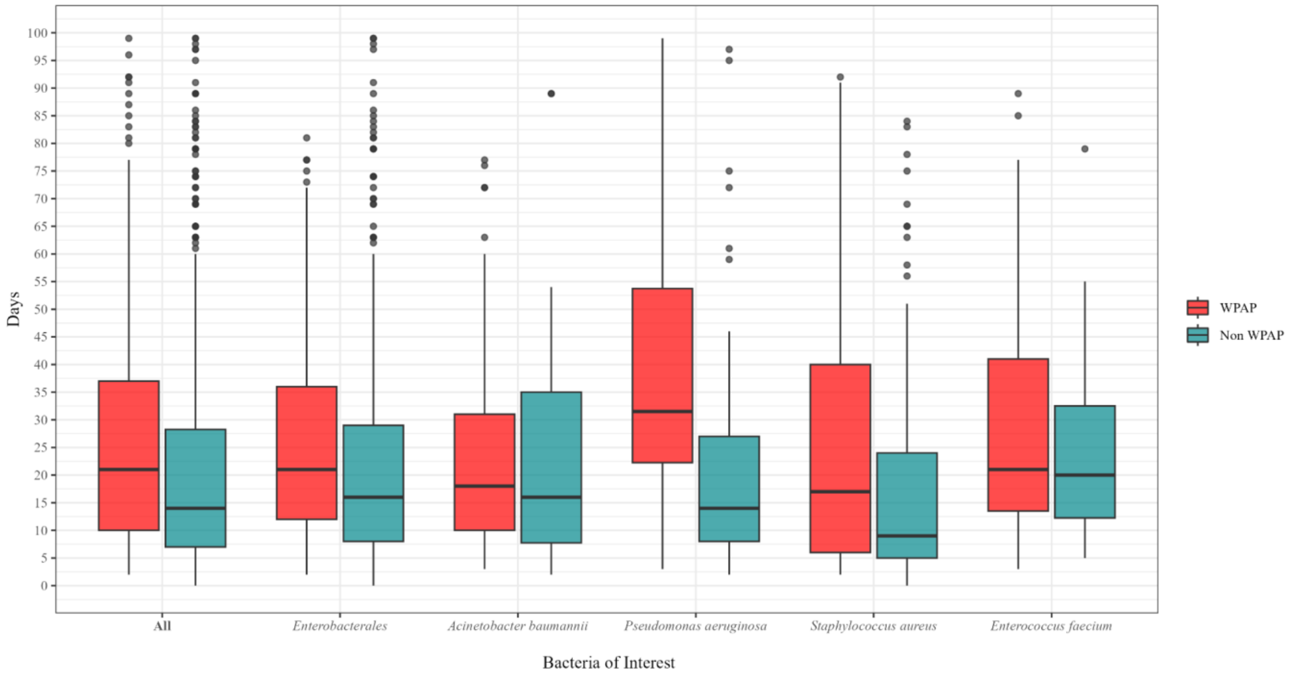

Supplementary Figure 1. Length of hospitalisation stay before recovery of each bloodstream infection isolates.

WPAP, WHO priority antimicrobial resistance phenotype. Median (IQR) length of hospital stay (days): All WPAP versus non-WPAP = 21 (IQR, 10·0-37·0) and 14 (7·0-28·8), respectively, p<0·001; carbapenem-resistant *Enterobacterales* versus carbapenem-susceptible *Enterobacterales* = 21·0 (12·0-36·0) and 16 (8·0-29·0), respectively, p=0·01; carbapenem-resistant *A. baumannii* versus carbapenem-susceptible *A. baumannii*= 18 (10·0-31·0) and 16 (7·8-35·0), respectively, p=0·38; carbapenem-resistant *P. aeruginosa* versus carbapenem-susceptible *P. aeruginosa* = 31·5 (22·2-53·8) and 14 (8-28), respectively, p<0·001; methicillin-resistant *S. aureus* versus methicillin-susceptible *S. aureus*= 17 (6·0-40·0) and 9 (5·0-24·0), respectively, p=0·01*;* and vancomycin-resistanct *E. faecium* versus vancomycin-susceptible *E. faecium* =21 (13·5-41) and 20 (12·2-32·5), respectively, p=0·25.

Supplementary Table 5. Incidence-density of all bacteria of interest in hospitalised patients.

	All bacteria		WPAP		
	n	Incidence Rate (95% CI), n/ 1000 patients-day*	n	Incidence Rate (95% CI), n/ 1000 patients-day*	Incidence-density Ratio (95% CI); p †
All	1200	1·91 (1·81 - 2·02)	480	0·77 (0·70-0·84)	NA
Enterobacterales	671	1·07 (0·99 - 1·15)	235	0·37 (0·33 - 0·43)	NA
Klebsiella pneumoniae	295	0·47 (0·42 - 0·53)	170	0·27 (0·23 - 0·32)	NA
Acinetobacter baumannii	142	0·23 (0·19 - 0·27)	124	0·20 (0·16 - 0·24)	NA
Pseudomonas aeruginosa	103	0·16 (0·13 - 0·20)	31	0·05 (0·03 - 0·07)	NA
Staphylococcus aureus	222	0·35 (0·31 - 0·40)	55	0·09 (0·07 - 0·11)	NA
Enterococcus faecium	62	0·10 (0·08 - 0·13)	35	0·06 (0·04 - 0·08)	NA
**ICU** §					
All	437	5·80 (5·27-6·37)	234	3·11 (2·72 - 3·53)	7·00 (5·85-8·37); p<0·0001
Enterobacterales	234	3·11 (2·72 - 3·53)	116	1·54 (1·27 - 1·85)	7·16 (5·54-9·25); p<0·0001
Klebsiella pneumoniae	117	1·55 (1·29 - 1·86)	82	1·09 (0·87 - 1·35)	6·86 (5·06-9·24); p<0·0001
Acinetobacter baumannii	70	0·93 (0·73 - 1·17)	66	0·88 (0·68 - 1·12)	8·36 (5·87-11·92); p<0·0001
Pseudomonas aeruginosa	43	0·57 (0·41 - 0·77)	14	0·19 (0·10 - 0·31)	6·04 (2·93-12·26); p<0·0001
Staphylococcus aureus	52	0·69 (0·52 - 0·91)	18	0·24 (0·14- 0·38)	3·57 (1·99-6·18); p<0·0001
Enterococcus faecium	38	0·51 (0·36 - 0·69)	20	0·27 (0·16 - 0·41)	9·79 (5·03-19·43); p<0·0001
**Non-ICU**§					
All	763	1·38 (1·29-1·48)	246	0·45 (0·39 - 0·51)	Reference group of IDR
Enterobacterales	437	0·79 (0·72 - 0·87)	119	0·22 (0·178 - 0·258)	Reference group of IDR
Klebsiella pneumoniae	178	0·32 (0·28 - 0·37)	88	0·16 (0·13 - 0·20)	Reference group of IDR
Acinetobacter baumannii	72	0·13 (0·10 - 0·16)	58	0·11 (0·08- 0·14)	Reference group of IDR
Pseudomonas aeruginosa	60	0·11 (0·08 - 0·14)	17	0·03 (0·02 - 0·05)	Reference group of IDR
Staphylococcus aureus	170	0·31 (0·26 - 0·36)	37	0·07 (0·05 - 0·09)	Reference group of IDR
Enterococcus faecium	24	0·04 (0·03 - 0·07)	15/	0·03 (0·02 - 0·05)	Reference group of IDR

WPAP, WHO priority antimicrobial resistance phenotype; n, number of WPAP isolates; N, number of all bacteria of interest isolates; CI, Confidence interval; IDR, incidence-density ratio; NA not applicable; CRE, carbapenem-resistant *Enterobacterales*; CRKP, carbapenem-resistant *Klebsiella pneumoniae* complex; CRAB, carbapenem-resistant *Acinetobacter baumannii* complex; CRPA, carbapenem-resistant *Pseudomonas aeruginosa*; MRSA, methicillin-resistant *Staphylococcus aureus*; VRE, vancomycin-resistant *Enterococcus faecium*; ICU, Intensive care unit. * Two hospitals that did not provide the number of patients-day were excluded from the analysis: total number of patients-day = 627,779 (75,302 ICU patients-day and 552,477 non-ICU patients-day). †IDRs refer to the comparison of incidence rate of WPAP episodes in ICU and non-ICU patients (reference).

Supplementary Table 6. Proportion of WPAP among each *Enterobacterales, Acinetobacter baumannii* complex, *Pseudomonas aeruginosa, Staphylococcus aureus* and *Enterococcus faecium* isolates.

	n/N	% (95% CI)
**All**	NA	NA
CRE	260/763	34·1 (26·5-42·5)
CRKP	192/332	57·8 (48·0-67·0)
CRAB	129/147	87·8 (79·3-93·1)
CRPA	32/120	26·7 (18·4-36·9)
MRSA	66/255	25·9 (17·9-35·8)
VRE	37/65	56·9 (44·8-68·3)
**ICU †**	NA	NA
CRE	126/277	45·5 (31·4-60·4)
CRKP	90/133	67·7 (51·1-80·7)
CRAB	68/72	94·4 (83·9-98·2)
CRPA	15/49	30·6 (18·0-47·1)
MRSA	21/59	35·6 (21·8-52·3)
VRE	20/38	52·6 (42·7-62·4)
**non-ICU †**	NA	NA
CRE	134/486	27·6 (22·2-33·7)
CRKP	102/199	51·2 (42·8-59·7)
CRAB	61/75	81·3 (71·9-88·1)
CRPA	17/71	23·9 (14·3-37·3)
MRSA	45/196	23·0 (15·7-32·3)
VRE	17/27	63·0 (43·3-79·1)

WPAP, WHO priority antimicrobial resistance phenotype; IDR, Incidence-density ratio; CI, Confidence interval; NA, not applicable; CRE, carbapenem-resistant *Enterobacterales*; CRKP, carbapenem-resistant *Klebsiella pneumoniae* complex; CRAB, carbapenem-resistant *Acinetobacter baumannii* complex; CRPA, carbapenem-resistant *Pseudomonas aeruginosa*; MRSA, methicillin-resistant *Staphylococcus aureus*; and VRE, vancomycin-resistant *Enterococcus faecium*; ICU, Intensive care unit; NE, not evaluated due to the small number of isolates.

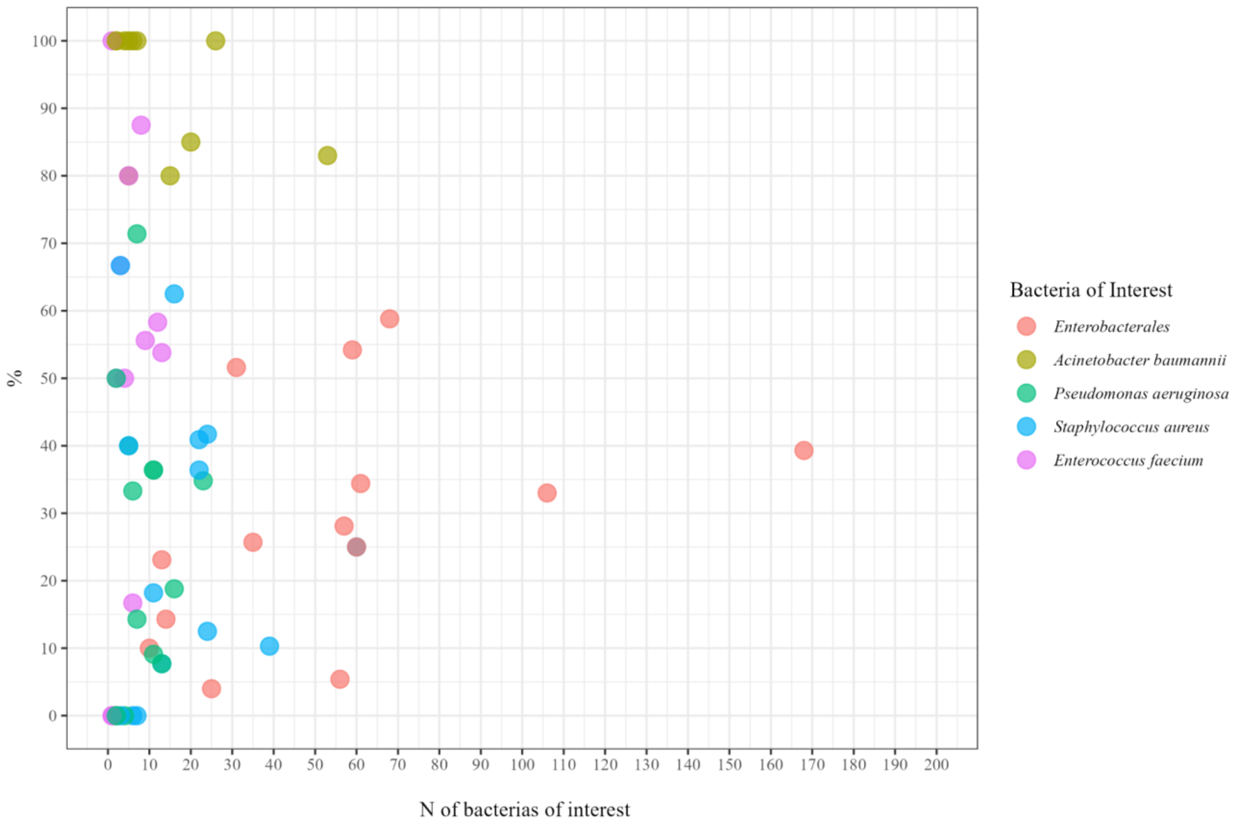

Supplementary Figure 2. Number of isolates and proportion of WPAP among each bacteria of interest by hospital.

WPAP, WHO priority antimicrobial resistance phenotype. Each circle represents one hospital. The proportion of carbapenem resistance in each hospital ranged from 0 to 100%, 4 to 59% and 0 to 71% in *A. baumannii*, *Enterobacterales* and *P. aeruginosa*, respectively; while the proportion of methicillin-resistant *S. aureus* and vancomycin-resistant *E. faecium* ranged from 0 to 67% and 0 to 88%, respectively.

Supplementary Table 7. Isolates that were identified as different species by MALDI-ToF at the central laboratory.

Automated system identification *	MALDI-ToF identification
Serratia liquefaciens	*Serratia marcescens*
Serratia marcescens	*Serratia ureilytica*

* Vitek-2® or BD Phoenix®.

Supplementary Table 8. Results of the quantitative multiplex polymerase chain followed by high-resolution melting analysis for carbapenemase genes* of carbapenem-resistant *Enterobacterales* species.

**Carbapenem-resistant *Enterobacterales***	Total tested n= (%)	bla_{KPC} n (%)†,	bla_{NDM} n (%)†	bla_{KPC} + bla_{NDM} n (%)†	None n (%)
All	197	125	55	14	3
Klebsiella pneumoniae complex	147	102 (69·4)	31 (21·1)	12 (8·2)	2 (1·4)
Serratia marcescens	21	16 (76·2)	3 (14·3) ‡	1 (4·8)	1 (4·8)
Proteus mirabilis	6	1 (16·7)	5 (83·3)	0	0
Enterobacter cloacae complex	7	3 (42·9)	4 (57·1)	0	0
Providencia stuartii	3	0	2 (66·7)	1 (33·3)	0
Serratia ureilytica	3	1 (33·3)	2 (66·7)§	0	0
Proteus penneri	2	0	2 (100)	0	0
Morganella morganii	2	0	2 (100)	0	0
Klebsiella oxytoca	1	1 (100)	0	0	0
Klebsiella aerogenes	1	1 (100)	0	0	0
Citrobacter freundii	1	0	1 (100)	0	0
Citrobacter koseri	1	0	1 (100)	0	0
Escherichia coli	1	0	1 (100)	0	0
Providencia rettgeri	1	0	1 (100)	0	0

CI, Confidence interval. * bla_{KPC}, bla_{NDM}, $bla_{OXA\text{-}48\text{-}like}$, bla_{IMP}, bla_{GES} and bla_{VIM}. † Percentual and 95% CI refers to the species. ‡ One isolate also carried bla_{GES}.

References

1 Monteiro J, Widen RH, Pignatari ACC, Kubasek C, Silbert S. Rapid detection of carbapenemase genes by multiplex real-time PCR. *J Antimicrob Chemother* 2012; 67: 906–9.

2 Woodford N, Ellington MJ, Coelho JM, *et al.* Multiplex PCR for genes encoding prevalent OXA carbapenemases in Acinetobacter spp. *Int J Antimicrob Agents* 2006; 27: 351–3.

3 Zeileis A. Object-oriented Computation of Sandwich Estimators. *J Stat Soft* 2006; 16: 1–16.

4 Cummings P. Analysis of Incidence Rates. CRC Press, 2019.
