# Supplementary material for: World Health Organization priority antimicrobial resistance in Enterobacterales, Acinetobacter baumannii, Pseudomonas aeruginosa, Staphylococcus aureus and Enterococcus faecium healthcare-associated bloodstream infections in Brazil (ASCENSION): a prospective, multicentre, observational study
Source: Lancet Reg Health Am. 2025 Jan 30;43:101004. doi: 10.1016/j.lana.2025.101004 (PMC11830303; doi:10.1016/j.lana.2025.101004)
Supplement: List of ASCENSION Study Group [file mmc4.docx]

| **ASCENSION Study Group** | |
| --- | --- |
| **First and middle name** | **Surname** |
| Jéssica Nesello | dos Santos |
| Charles Francisco | Ferreira |
| Tarsila | Vieceli |
| Julival Ribeiro | Fagundes |
| Raquel Nascimento | Matias |
| Shisue Karina | Katagiri |
| Olavo José Vicente | Neto |
| Rafaela Kuczynski | da Rocha |
| Claudia Maria Dantas de Maio | Carrilho |
| Mila Muraro | de Almeida |
| Heloisa da Silva | Rosa |
| Valéria Paes | Lima |
| Tazio | Vanni |
| Simone Aranha | Nouer |
| Elizabeth Mendes | Alves |
| Jorge Luiz Nobre | Rodrigues |
| André Jhonathan | Dantas |
| Gyselle de Souza | Rebouças |
| Jailton Santos | de Oliveira |
